# Safety and early outcomes of simultaneous bilateral TKA in patients with BMI > 40: A retrospective comparative study

**DOI:** 10.1051/sicotj/2025019

**Published:** 2025-04-14

**Authors:** Alexandre Le Guen, Zakee Azmi, Jesper Fritz, Aymen Alqazzaz, Sébastien Parratte

**Affiliations:** 1 Department of Orthopaedic Surgery and Trauma, Hôpital Pierre Paul Riquet Place du Dr Baylac – TSA 40031 31059 Toulouse cedex 9 France; 2 International Knee and Joint Centre Hazza Bin Zayed St. P.O. Box 46705 Abu Dhabi United Arab Emirates; 3 Department of Orthopedic Surgery, University of Pennsylvania Health System Philadelphia Pennsylvania

**Keywords:** Obesity, Bilateral total knee arthroplasty, Middle East, Osteoarthritis

## Abstract

*Introduction*: Simultaneous bilateral total knee arthroplasties (SBTKA) are common in Asia, but surgeons may have a body mass index (BMI) threshold for performing these procedures. However, no guidelines regarding patient weight and SBTKA exist in the literature. We hypothesized that SBTKA can be performed safely and efficiently for morbidly obese patients. We aimed to compare 1) the rate of complications within one year after surgery, 2) operative time, blood loss, and length of stay, and 3) clinical outcomes at one year after SBTKA in patients with BMI < 30 versus 30 < BMI < 40 and BMI > 40. *Methods*: In this retrospective comparative matched (age, ASA score) study, we evaluated 113 patients who underwent SBTKA (posterior stabilized cemented TKA), between 2019 and 2022. The patient population was grouped based on their BMI: BMI < 30 (33 patients), 30 < BMI < 40 (43 patients), and BMI > 40 (37 patients). A complication was defined as an event that could be classified as a grade > 3 according to the Clavien-Dindo classification within one year of surgery. Data on complication rate, operation time, blood loss, and preoperative and post-operative function KSS at one year were compared. *Results*: No significant difference in the occurrence of early complications between the three groups was observed. One patient was readmitted for periprosthetic fracture in the BMI < 30 group. There was no significant difference in operative time, blood loss, and KSS score at one year between the three groups. A significant functional improvement was observed in all three groups at the one-year follow-up. *Discussion*: This study suggests that SBTKA in patients with a BMI > 40 is safe, with no increased complications, similar surgical time, and blood loss. Significant functional improvement was observed at one year postoperatively. While promising, further multi-center studies are needed to confirm these findings and evaluate long-term outcomes.

## Introduction

Total knee arthroplasty (TKA) is the treatment of choice for advanced knee degenerative joint disease (DJD) that has failed conservative treatment. Although DJD presents as a unilateral joint disorder in the majority of cases, 20% of patients in the United States suffer from advanced bilateral DJD, necessitating bilateral TKA [1]. Notably, among those who undergo unilateral TKA, approximately 30% of patients experience exacerbation of contralateral knee pain within 6 months postoperatively [2], and nearly 40% develop pain and functional limitations in both knees, requiring contralateral TKA within 8 years [3]. Furthermore, patients with known symptoms in the contralateral knee following unilateral TKA have been shown to have poor functional improvement [4]. Consequently, simultaneous bilateral TKA (SBTKA) has emerged as a viable option to for patients with bilateral knee disease.

The prevalence of bilateral knee DJD in Asia is reported to be as high as 50%, surpassing rates observed in Western countries [5]. These regional disparities suggest that environmental, ethnic, and/or genetic factors may significantly influence the development of bilateral knee DJD. Obesity is a recognized risk factor for bilateral knee DJD [6]. Globally, over 502 million people are affected by obesity [7], with the Middle East reporting particularly high rates – where in some countries, more than 50% of the population is classified as either overweight or obese [8]. Projections indicated that these trends will continue to rise, with higher obesity rates by 2030 [9]. Furthermore, obesity and morbid obesity, defined as body mass index (BMI) ≥ 30 and ≥ 40 kg/m^2^ respectively, present a unique challenge to orthopaedic surgeons, due to their associations with increased operative times, extended hospital stays, and an increased incidence of infection and major postoperative complications [10, 11]. The risk factors make it especially challenging for surgeons considering SBTKA for morbidly obese patients.

There is a paucity of literature examining SBTKA and outcomes in morbidly obese patients. Therefore, we set out to: 1) compare the complication rate in morbidly obese patients undergoing SBTKA within one year following the procedure using the Clavien-Dindo classification [12], 2) compare the operative time, blood loss and length stay and 3) compare the clinical outcome scores preoperatively and at one year using the KSS score [13].

## Material and methods

After institutional review board approval, this retrospective comparative study was conducted on patients who underwent a simultaneous bilateral total knee arthroplasty between January 2019 and December 2022 by the same surgeon (S.P.) in Abu Dhabi. The sample size of the study was calculated to be at least 80 patients in the trial to obtain 80% power to detect a difference in the primary endpoint.

**Figure 1:**
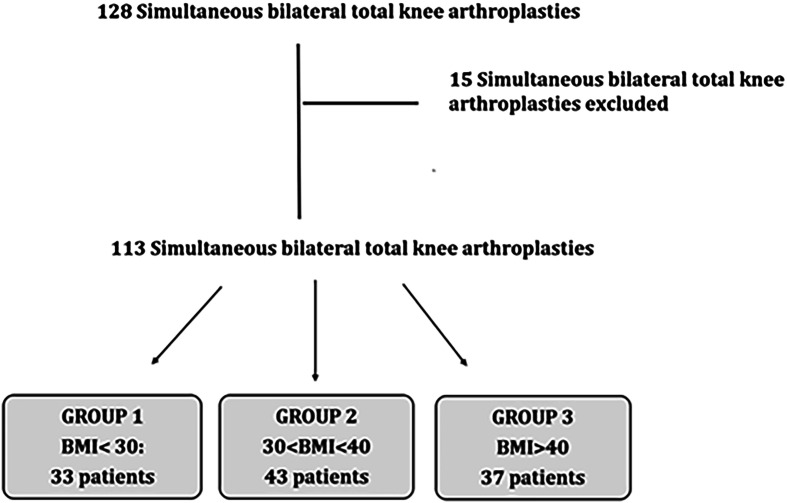
Flow diagram of patient selection for the analyses.

Inclusion criteria were age > 18 years and candidates for simultaneous bilateral TKA for the treatment of end-stage knee disease secondary to degenerative or post-traumatic arthritis. Osteoarthritis was diagnosed through physical examination and radiological imaging. Exclusion criteria included: Sequential bilateral TKA, TKA with extensor mechanism repair, revision TKA, UKA, history of prior post-traumatic extra-articular deformity exceeding 10 degrees and severe neurological or musculoskeletal disorders. One hundred and twenty-eight simultaneous bilateral total knee arthroplasties were performed during the study period. Patients who agreed to participate in the study completed written informed consent. Fifteen were excluded for the above reasons. One hundred and thirteen were enrolled for evaluation during the study period and stratified into three groups based on their BMI status. This gave the following breakdown: Group 1 with BMI < 30 (*n* = 33), Group 2 with 30 < BMI < 40 (*n* = 43), and Group 3 with BMI > 40 (*n* = 37) (Figure 1).

Additionally, we followed the recommendations of the American Diabetes Association to stabilise glycaemic parameters and delay surgery if glycaemic control was not satisfactory, and to treat known comorbidities or those discovered during the preoperative assessment [14]. Thus, patients with diabetes had good control of HbA1c (7 mmol/L) to reduce the infectious risk [14].

**Figure 2:**
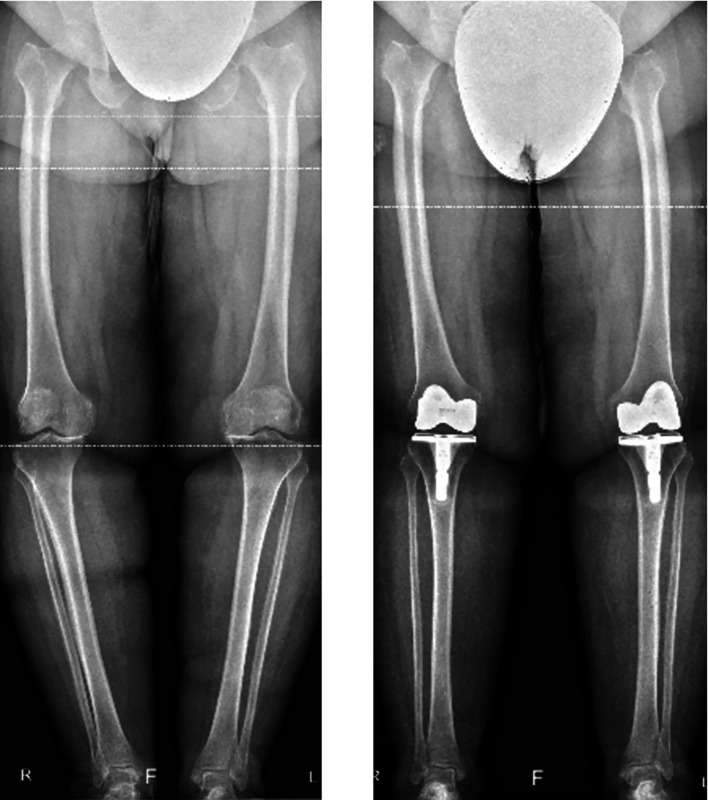
Preoperative and postoperative X-rays of a patient with a BMI of 42 who underwent bilateral total knee arthroplasty with a short tibial stem.

The surgical technique was standardized for all patients and performed by a single surgeon with subspecialty training and interest in TKA. A three-compartment cemented prosthesis, stabilized posteriorly and fitted with a mobile tray, was inserted using a subvastus approach and without a tourniquet. A short tibial stem was systematically used in patients with a BMI greater than 30 (Figure 2). To optimize preparation, a pulsed lavage was performed on the recipient bone. Intra-articular drains were not routinely used. Only sutures were used to close the surgical site.

Postoperatively, all patients received standard deep veinous thrombosis prophylaxis. Rehabilitation was standardized with full weight bearing with crutches and immediate mobilisation. The same anaesthetic protocol was used for all patients and was performed by a few selected anaesthetists.

All data were obtained from the clinic’s computerized database of patient records.

Patient data included gender, age, BMI, and ASA score. Except for gender, baseline demographic and clinical characteristics were well-balanced across the three groups (Table 1).

**Table 1:** Baseline characteristics of participants.

	Group 1	Group 2	Group 3	*P*-value
	BMI < 30	30 < BMI < 40	BMI > 40	
	*N* = 33	*N* = 43	*N* = 33	
BMI (kg/m^2^)				
Mean (SD)	26.5 (2.7)	33.0 (1.9)	40.2 (3.5)	<0.0001
Gender, *N* (%)				
Women	21 (63.6)	26 (60.4)	33 (89.1)	0.009
Men	12 (36.4)	17 (39.6)	4 (10.9)	
Age (years)				
Mean (SD)	63.6 (11.0)	63.2 (9.1)	61.8 (6.1)	0.666
ASA score				
Median (SD)	2 (0.6)	2 (0.5)	2 (0.4)	0.949

Complications within a year of surgery and their rates were assessed from the patient records. Only complications of grade 3 or higher on the Clavien-Dindo scale were recorded, defined as any event outside the usual post-operative course requiring either readmission or return to the operating theatre [15]. Operative time was calculated from start and end times taken from operative notes completed by surgical nursing staff as part of the patient’s medical record. Blood loss was calculated using the Orthopedic Surgery Transfusion Hemoglobin European Overview (OSTHEO) formula [16]. Hematocrit and hemoglobin levels were taken the day before surgery and the first day after surgery. Length of hospital stay was measured from the date of admission to the date of discharge. All patients were admitted by afternoon before the day of surgery.

Clinical outcome at one year was assessed using Knee Society Scores (KSS). These scores were collected from patient-completed questionnaires and by the operating surgeon. Preoperative scores were collected at the time of the last consultation before surgery. Thereafter, scores were assessed at biannual intervals during consultations. The difference between pre-operative scores and scores collected at one year postoperatively was used as a signifier of clinical improvement.

Data are presented as mean values associated with standard deviation. Categorical outcomes were compared using Fisher’s exact test and chi-squared test. Normally distributed continuous variables were compared using Student *t*-test or ANOVA. The threshold for statistical significance were based on a two-sided *p*-value < 0.05. Statistical calculations were performed with RStudio IDE (version 2024.04.2) using R (4.4.0) programming language.

## Results

No statistically significant difference across the three groups in the rate of complications equal or exceeding 3 in the Clavien-Dindo scale was found (*p* = 0.149) (Table 2). Only one major complication was reported in the study population. On postoperative day 12, one patient in group 1 (BMI < 30) suffered a periprosthetic fracture of the distal femur. Percutaneous screw fixation was performed on day 19. A new revision of the left TKA using a distal segmental femur was performed on postoperative day 42. The same patient developed a haematoma requiring aspiration after a fall on day 74, which was attributed to pre-existing orthostatic hypotension. Examination and imaging revealed a post-traumatic dislocation of the patella with rupture of the medial retinaculum. On day 101, the patient underwent an Insall procedure with reduction of the patella and release of the lateral retinaculum.

**Table 2:** Overview of per and postoperative results between the three groups.

	Group 1	Group 2	Group 3	*P*-value
	BMI < 30	30 < BMI < 40	BMI > 40	
	*N* = 33	*N* = 43	*N* = 33	
AIM 1				
Number of complications	1	0	0	0.149
AIM 2				
Surgical Time (Min)	147.0 (20.6)	149.1 (21.9)	145.0 (15.6)	0.712
Mean (SD)				
Blood loss (mL)	166.7 (86.7)	171.9 (111.8)	178.3 (87.0)	0.883
Mean (SD)				
Length of stay (days)	5.1 (1.8)	5.1 (2.2)	4.9 (2.05)	0.883
Mean (SD)				
AIM 3	50.7 (8.6)	52.0 (12.2)	57.4 (8.6)	0.07
KSS difference				
Mean (SD)				

Concerning the mean operating time for simultaneous bilateral total knee arthroplasty, there was no statistically significant difference between the means of the three groups (*p* = 0.712).

Concerning blood loss, as measuring blood loss during surgery potentially underestimates losses by around 50% we use the OSTHEO formula. No statistically significant difference was detected between the means of the three groups (*p* = 0.883). The mean length of stay was 5 days across all groups. No statistically significant difference was detected between the means of the three groups. (*p* = 0.883). A one-way ANOVA revealed that there was no statistically significant difference in KSS at one year between the three groups (*p* = 0.070). All groups showed significant improvement at one year compared to pre-surgery, as evidenced by increases in all scores. All the results are presented in Table 2.

## Discussion

Obesity is increasingly prevalent in the Middle East, and the utilization of SBTKA is common in Asia. Stemming from our clinical experience and the results of some studies [17], we hypothesized that SBTKA can be performed in morbidly obese patients without major early complications in a Middle East population. Including 113 patients undergoing bilateral posterior stabilized cemented TKA, we found no statistically significant difference in major adverse events across different BMI groups in the first year postoperatively. There was also no difference in operative time, blood loss, length of stay, and clinical outcomes at one year between the three groups.

A meta-analysis by Kerkhoffs et al. demonstrated obese patients undergoing TKA to be at higher risk for complications, including infections and revision [18]. Conversely, our study demonstrated that obesity was not a risk factor for early complications. Notably, these results must be analyzed in light of the United Arab Emirates’ sociocultural context. All preoperative and postoperative patient management, dressing care, and physiotherapy were carried out at the main center. Patients were surrounded by medical and paramedical staff accustomed to caring for obese and morbidly obese patients who have undergone total knee arthroplasty. Similarly, Turki et al., in a retrospective study in Saudi Arabia, reported a low rate of surgical site infection in obese and morbidly obese patients [19]. We believe that the geographically-specific factors may contribute to low infection rates and require further investigation.

Furthermore, we did not find any differences in terms of operating time, blood loss, and length of stay. All patients were operated on by the same experienced surgeon and a trained team, which may contribute to efficiency and reduction of operative times. Our operative time was similar to that reported in the literature and was not affected by obesity or morbid obesity [20]. This is critical to highlight since a shorter operative time is a protective against postoperative infection [21]. A similar surgical time in morbidly obese patients is consistent with other studies and important to consider when adapting practice setting and resources [19]. Using the OSTHEO formula, blood loss was related to patients’ BMI in this study and was lower to other publications [16]. As a result, morbidly obese patients in our study were not at higher risk for post-operative transfusions. This is in contrast to publications comparing bilateral versus staged TKA [22]. Length of stay was also similar between the three groups and similar to the literature, averaging 4 to 5 days [23]. Recent evidence suggests that early discharge from the acute care unit reduces the risk of hospital-acquired complications such as nosocomial infections and thromboembolic events [23]. Thus, we believe early appropriate discharge must be emphasized in all patients, and morbid obesity alone, barring other risk factors or complications, should not preclude that.

At the one-year postoperative follow-up, all patients, including those with morbid obesity, showed a significant improvement in their functional scores. Most patients achieved a KSS ≥ 90, indicating excellent outcomes [24]. This improvement in KSS suggests that patients can restore near-normal knee function, potentially lowering the risk of future disability and improving overall quality of life [24]. These findings reinforce that, with proper surgical intervention and rehabilitation, obese and morbidly obese patients can achieve significant functional recovery without increased for complications [20].

Our study has several limitations. It is inherently subject to the limitations of retrospective, single institution, single surgeon study. The number of morbidly obese patients was limited to thirty seven. Additionally, the potential for unobserved confounders and idiosyncrasies in surgeon technique or environmental factors specific to our institution may have influenced our results. Additionally, differences in fat distribution were not graded. Central versus gluteofemoral fat deposition patterns may predict the difficulty of surgical exposure and potentially alter outcomes [25].

Our follow-up was limited to one year after surgery. Although Kerkhoffs et al. showed in a meta-analysis that obese patients treated with TKA had more complications, mainly in the short term (<1 year), follow-up should be continued to study mid- and long-term complications such as septic and aseptic loosening over time [18]. It may also be interesting to investigate whether tibial fixation reduces the long-term risk of mechanical loosening in obese patients. Further studies are warranted in obese patients who have undergone SBTKA to better assess survivorship and function. Despite these limitations, the present study was one of the few to investigate the complications and outcomes of bilateral simultaneous knee replacement in morbidly obese patients.

The results of this study, therefore, adds to a growing body of evidence suggesting that obesity and morbid obesity alone should not be a contraindication for SBTKA [19]. Implementing lower BMI cutoffs in the Middle East could lead to the unnecessary exclusion of a significant number of patients from surgical care, despite evidence suggesting no increased risk of complications. We suggest a holistic assessment and shared decision-making instead of an appropriate target weight reduction for Middle Eastern patients.

## Conclusion

This study showed no increase rate of major complication at one year for patients with a BMI > 40 with similar surgical time, blood loss, and length of stay. A significant improvement in functional scores compared to pre-surgery was observed at one year. Therefore, SBTKA for patients with BMI > 40 is possible in the Middle East seems possible in the setting of an experienced practice and team. While promising, further multi-center studies are needed to confirm these findings and evaluate long-term outcomes.

## Data Availability

The datasets used and/or analysed during the current study are available from the corresponding author on reasonable request.
